# Editorial for the Special Issue “Machine Learning in Computer Vision and Image Sensing: Theory and Applications”

**DOI:** 10.3390/s24092874

**Published:** 2024-04-30

**Authors:** Subrata Chakraborty, Biswajeet Pradhan

**Affiliations:** 1 School of Science and Technology, University of New England, Armidale, NSW 2351, Australia; 2 Centre for Advanced Modelling and Geospatial Information Systems (CAMGIS), School of Civil and Environmental Engineering, Faculty of Engineering & IT, University of Technology Sydney, Sydney, NSW 2007, Australia; biswajeet.pradhan@uts.edu.au; 3 Griffith Business School, Griffith University, Nathan, QLD 4111, Australia; 4 Earth Observation Centre, Institute of Climate Change, Universiti Kebangsaan Malaysia (UKM), Bangi 43600, Selangor, Malaysia

Machine learning (ML) models have experienced remarkable growth in their application for multimodal data analysis over the past decade [1]. The diverse applications of ML models span domains such as medical image [2,3,4] and signal processing [5,6], remote sensing for earth observation and monitoring [7,8,9], the detection of daily human activities [10,11], and many more. ML models play a significant role in supporting computer vision and image-sensing applications, helping to unravel complex and real-world challenges. Recent developments in ML empower us to better analyse image and sensor data, motivating extensive research initiatives aimed at addressing applied challenges in multiple domains, including healthcare, agriculture, defence, remote sensing, earth observation, and autonomous navigation.

This Special Issue aims to compile a compendium of high-quality research addressing the broad challenges in both the theoretical and applied aspects of advanced ML models in the field of computer vision and image sensing. The 11 papers accepted in this Special Issue encompass key original research in areas such as medical applications, earth observation, and human detection, alongside comprehensive review papers on the application of ML in imaging and sensing.

Contribution 1 developed non-deep active learning models capable of significantly improving the annotation efficiency of unlabelled images. While conventional deep neural network-based approaches often require a large number of computation nodes and extensive computation time when selecting the most informative unlabelled images, the proposed method first trains a task model on labelled images to predict unlabelled ones. An uncertainty indicator is then generated for each unlabelled image, with images exhibiting a high uncertainty index nominated for annotation due to their information richness. The proposed method outperforms the current SoTA method by 1% accuracy on CIFAR-10.

Contribution 2 developed the unique River Obstacle Segmentation En-Route by USV Dataset (ROSEBUD), which is assessable for public use in robotic SLAM applications to map both water and non-water objects and obstructions in fluvial images from the water level. ROSEBUD provides an exciting baseline dataset applicable for complex surface navigation through intricate fluvial scenes. The dataset comprises 549 diverse images, including variations in water quality, seasons, and obstacle types, manually annotated and obtained from narrow inland water bodies. Two state-of-the-art networks trained on existing water segmentation datasets were tested for generalisation to the ROSEBUD dataset. While modern custom networks designed for water recognition in marine images can successfully segment large areas, they may encounter challenges with small obstacles in fluvial scenes unless specifically retrained on the ROSEBUD dataset.

Contribution 3 developed a novel Alzheimer’s classification approach. Alzheimer’s disease is a chronic brain ailment that adversely affects language, orientation, bodily functions, memory, and cognitive ability, among other aspects. Imaging plays a crucial role in the diagnosis of Alzheimer’s disease, and deep learning has emerged as a key tool for image processing and classification in this context. In this study, a deep learning model has been developed for early diagnosis from MRI images using transfer learning and Gorilla Troops optimisation. The proposed framework, A3C-TL-GTO, is designed for MRI image classification, specifically for detecting Alzheimer’s. The A3C-TL-GTO framework is developed and evaluated using both the Alzheimer’s dataset (with four classes) and the Alzheimer’s Disease Neuroimaging Initiative (ADNI) datasets. The new framework aims to reduce bias and variability in preprocessing steps and hyperparameter optimisation for the classifier model. Experimental outcomes demonstrate that the proposed framework achieves 96.65% accuracy for the Alzheimer’s Dataset and 96.25% accuracy for the ADNI dataset, surpassing current state-of-the-art methods.

Contribution 4 developed a novel human detection approach that combines a pretrained human face detector based on a multitask cascaded convolutional neural network with a traditional pedestrian detector based on aggregate channel features, employing a score combination module. The model is designed to achieve pedestrian detection in scenarios with limited datasets and computational resources. The robustness of the proposed detector was tested through cross-dataset validations on various pedestrian datasets, including INRIA, part of ETHZ, Caltech, and Citypersons. Experiments demonstrated that the integrated detector significantly improves recall and reduces the log-average misclassification rate compared to the traditional pedestrian detector used alone. Simultaneously, the proposed method achieves performance equivalent to FRCNN on the INRIA test set compared to the use of the Aggregated Channel Features detector alone.

Contribution 5 developed a deep learning neural network model to accurately detect regions of pneumothoraxes in chest X-ray images. The model integrates a Mask Regional Convolutional Neural Network (Mask RCNN) and transfer learning with ResNet101 as a backbone feature pyramid network (FPN). The proposed model was trained and tested on a dataset prepared by the Society for Imaging Informatics in Medicine in association with the American College of Radiology (SIIM-ACR). The present work compares the performance of the proposed MRCNN model, which is based on ResNet101 as an FPN, with the conventional model based on ResNet50 as an FPN. The proposed model exhibited a significantly lower classification loss, bounding box errors, and mask errors compared to the conventional model based on ResNet50 as an FPN.

Contribution 6 developed a new method to enhance real-time semantic segmentation for autonomous driving. Given the growing interest in autonomous driving, achieving real-time semantic segmentation has become a necessary and popular challenge in computer vision. However, when deploying deep learning models to edge devices in vehicles, a suitable approach is required to strike the right balance between accuracy and computing time. Despite significant progress in real-time semantic segmentation, a substantial gap compared to general semantic segmentation methods remains. In this study, a network architecture based on a dual encoder and a self-attention mechanism was proposed. Compared with preceding works, this study achieved a 78.6% mIoU with a speed of 39.4 FPS at a resolution of 1024 × 2048 on a Cityscapes test submission.

Contribution 7 proposed an iris liveness detection (ILD) method to counteract spoofing attacks. This method leverages the global-level features of Thepade’s Sorted Block Truncation Coding (TSBTC) and the local-level features of the Gray-Level Co-occurrence Matrix (GLCM) from iris images. Thepade’s SBTC captures colour texture content as global features, while the GLCM extracts fine-texture features locally. The combination of global and local features aids in distinguishing between live and non-living iris samples. The extracted features are used to train nine leading ML classifiers, including naïve Bayes (NB), decision tree (J48), support vector machine (SVM), random forest (RF), and multilayer perceptron (MLP) classifiers, and several ensembles (SVM + RF + NB, SVM + RF + RT, RF + SVM + MLP, J48 + RF + MLP) for ILD testing. The experiment, which was conducted on four benchmark datasets, demonstrated improved results with the feature fusion approach. The new fusion approach achieved 99.68% accuracy using the RF + J48 + MLP ensemble of classifiers, closely followed by the RF algorithm, which attained 95.57%. Iris liveness detection holds promise for significantly enhancing human–computer interaction and identity security in the cyber–physical space.

Contribution 8 introduced a novel hybrid approach that combines deep learning with decision tree-based models. The new model presents predictions of lung cancer malignancy as visually interpretable decision trees. The deep learning component of the model is trained using a large, publicly available X-ray dataset on lung cancer. These models are then employed to infer biomarker scores for chest X-ray images from two independent data sets, both containing malignancy metadata. Multi-variate predictive models were subsequently derived by fitting shallow decision trees to the stratified malignancy datasets. The performance of these models was assessed using a range of metrics to identify the best model. The optimal decision tree model in this study achieved a sensitivity of 86.7%, a specificity of 80.0%, and a positive prediction score of 92.9%.

Contribution 9 compiled and compared the most recent developments in Artificial Intelligence (AI)-based techniques for diagnosing and classifying Amniotic Fluid Volume (AFV) levels. Additionally, a systematics breakdown of the factors and causes of abnormal AFV levels is presented, including placental abnormalities, kidney or central nervous system issues, as well as other contributors, such as preterm birth or twin-to-twin transfusion syndrome. The study provides a brief overview of various ML and deep learning (DL) models, along with key datasets. Challenges and opportunities in this field are summarised, along with future research directions.

Contribution 10 conducted a systematic review of state-of-the-art AI techniques applied to X-ray, CT, and US images for detecting COVID-19. This study highlights various approaches, the significance of these research efforts, potential challenges, and future trends related to implementing an AI system for disease detection during the COVID-19 pandemic. The early and rapid detection of COVID-19 is challenging yet crucial in halting the speed of the spread of the SARS-CoV-2 virus. The prior validation of AI in various scientific fields has prompted researchers to address this problem further. Multimodal imaging analyses, including X-ray, computed tomography (CT), and ultrasound (US) analyses, applying AI techniques have played a significant role in curbing the COVID-19 outbreak through early diagnosis. This study provides a comprehensive breakdown of such efforts.

Contribution 11 provided a comprehensive review of the applications of DL methods in Parkinson’s disease (PD) detection. PD stands as the second most common neurodegenerative ailment, affecting over 6 million individuals globally. Medications can slow down PD progression in its early stages, making an early diagnosis crucial for better patient management and intervention. AI-based diagnostic methods are gaining attention from medical professionals. This study identified 63 key studies published between January 2011 and July 2021 that applied DL-based models for the automated diagnosis of PD. These models utilised various types of image and signal modalities, including brain analyses (SPECT, PET, MRI, and EEG) and motion variations (gait, handwriting, speech, and EMG). The review identifies the best-performing DL model reported for each modality and highlights limitations hampering the adoption of such AI tools in healthcare. The study suggests a new direction for further research on deep learning in the automated detection of PD.

In summary, the eleven papers in this Special Issue on “Machine Learning in Computer Vision and Image Sensing: Theory and Applications” showcase the remarkable progress achieved in the computer vision domain through the application of ML models. This Special Issue contributes to key application areas, including fundamental improvements in image segmentation (contribution 1), applications in fluvial (contribution 2) and land (contribution 6) navigation, security applications with human detection (contribution 4) and iris analysis (contribution 7), and medical diagnoses of Alzheimer’s (contribution 3), pneumothoraxes (contribution 5), and lung cancer (contribution 8). Additionally, this Special Issue includes three key review papers highlighting the applications of ML in Amniotic Fluid Volume detection (contribution 9), COVID-19 detection (contribution 10), and Parkinson’s disease (contribution 11) detection. The field is steadily advancing towards enabling AI tools for a multitude of computer vision applications. These proficiencies will hopefully lead to transformative applications in this field, and the studies presented in this Special Issue provide an exciting snapshot of that growth.
